# Supplementary material for: Metagenomic analysis of the dynamical conversion of photosynthetic bacterial communities in different crop fields over different growth periods
Source: PLoS One. 2022 Jul 14;17(7):e0262517. doi: 10.1371/journal.pone.0262517 (PMC9282544; doi:10.1371/journal.pone.0262517)
Supplement: S1 Table — (DOCX) [file pone.0262517.s001.docx]

**Table S1.** Quality filtering of the 35 samples.

| **Sample Name** | **Raw PE** | **Combined** | **Qualified** | **Nochime** | **Base(nt)** | **AvgLen(nt)** | **Q20** | **Q30** | **GC%** | **Effective%** |
| --- | --- | --- | --- | --- | --- | --- | --- | --- | --- | --- |
| Sa1 | 90,526 | 90,414 | 89,618 | 89,618 | 18,547,453 | 207 | 98.54 | 95.62 | 65.48 | 99.00 |
| Sa2 | 87,279 | 87,185 | 86,463 | 86,463 | 17,893,955 | 207 | 98.57 | 95.69 | 65.47 | 99.07 |
| Sa3 | 84,437 | 84,358 | 83,642 | 83,642 | 17,308,870 | 207 | 98.53 | 95.64 | 65.6 | 99.06 |
| Sa4 | 99,981 | 99,903 | 99,016 | 99,016 | 20,492,576 | 207 | 98.61 | 95.79 | 65.55 | 99.03 |
| Sb1 | 87,948 | 87,868 | 87,055 | 87,055 | 18,016,653 | 207 | 98.63 | 95.84 | 65.6 | 98.98 |
| Sb2 | 94,700 | 94,598 | 93,913 | 93,913 | 19,438,685 | 207 | 98.67 | 95.9 | 65.56 | 99.17 |
| Sb3 | 98,522 | 98,450 | 97,669 | 97,669 | 20,215,472 | 207 | 98.65 | 95.88 | 65.58 | 99.13 |
| Sb4 | 69,238 | 69,158 | 68,550 | 68,550 | 14,187,828 | 207 | 98.62 | 95.8 | 65.59 | 99.01 |
| Ca1 | 98,585 | 98,169 | 97,341 | 97,341 | 20,202,838 | 208 | 98.49 | 95.51 | 64.88 | 98.74 |
| Ca3 | 89,118 | 88,948 | 88,315 | 88,315 | 18,291,895 | 207 | 98.58 | 95.69 | 64.67 | 99.10 |
| Ca2 | 82,961 | 82,667 | 82,118 | 82,118 | 17,019,256 | 207 | 98.6 | 95.75 | 65.6 | 98.98 |
| Cb1 | 95,630 | 95,482 | 94,694 | 94,694 | 19,596,451 | 207 | 98.6 | 95.78 | 65.06 | 99.02 |
| Cb2 | 72,142 | 72,064 | 71,514 | 71,514 | 14,798,848 | 207 | 98.57 | 95.71 | 64.97 | 99.13 |
| Cb3 | 90,774 | 90,664 | 89,911 | 89,911 | 18,613,415 | 207 | 98.56 | 95.65 | 65.01 | 99.05 |
| Cb4 | 93,916 | 93,825 | 93,081 | 93,081 | 19,263,595 | 207 | 98.57 | 95.7 | 64.98 | 99.11 |
| Cc1 | 99,220 | 99,131 | 98,422 | 98,422 | 20,385,427 | 207 | 98.69 | 95.98 | 65.41 | 99.20 |
| Cc2 | 99,361 | 99,303 | 98,577 | 98,577 | 20,402,285 | 207 | 98.69 | 95.97 | 65.29 | 99.21 |
| Cc3 | 98,198 | 98,129 | 97,448 | 97,448 | 20,195,018 | 207 | 98.68 | 95.93 | 65.41 | 99.24 |
| Cc4 | 76,260 | 76,168 | 75,612 | 75,612 | 15,669,088 | 207 | 98.67 | 95.92 | 65.43 | 99.15 |
| Ta1 | 91,200 | 90,569 | 90,066 | 90,066 | 18,662,969 | 207 | 98.26 | 94.85 | 65.58 | 98.76 |
| Ta2 | 94,539 | 94,170 | 93,732 | 93,732 | 19,424,191 | 207 | 98.4 | 95.11 | 65.18 | 99.15 |
| Ta3 | 98,556 | 96,717 | 96,127 | 96,127 | 19,907,638 | 207 | 98.26 | 94.89 | 64.27 | 97.54 |
| Tb1 | 84,670 | 83,574 | 83,227 | 83,227 | 17,491,359 | 210 | 98.27 | 94.81 | 65.34 | 98.3 |
| Tb2 | 94,369 | 94,252 | 93,910 | 93,910 | 19,432,501 | 207 | 98.43 | 95.23 | 65.32 | 99.51 |
| Tb3 | 93,664 | 93,578 | 93,175 | 93,175 | 19,284,656 | 207 | 98.44 | 95.21 | 65.37 | 99.48 |
| Tb4 | 83,544 | 83,027 | 82,658 | 82,658 | 17,144,341 | 207 | 98.38 | 95.12 | 65.48 | 98.94 |
| Tc1 | 96,709 | 96,434 | 95,999 | 95,999 | 19,900,876 | 207 | 98.36 | 95.03 | 65.23 | 99.27 |
| Tc2 | 96,904 | 96,834 | 96,388 | 96,388 | 19,946,604 | 207 | 98.4 | 95.13 | 65.11 | 99.47 |
| Tc3 | 78,146 | 77,822 | 77,484 | 77,484 | 16,071,266 | 207 | 98.43 | 95.24 | 65.28 | 99.15 |
| Sc1 | 90,690 | 90,640 | 90,228 | 90,228 | 18,672,482 | 207 | 98.38 | 95.09 | 65.77 | 99.49 |
| Sc2 | 88,718 | 88,638 | 88,274 | 88,274 | 18,268,509 | 207 | 98.45 | 95.21 | 65.57 | 99.5 |
| Sc3 | 94,594 | 94,491 | 94,024 | 94,024 | 19,458,099 | 207 | 98.37 | 95.04 | 65.53 | 99.4 |
| Sc4 | 92,456 | 92,355 | 91,941 | 91,941 | 19,027,668 | 207 | 98.37 | 95.07 | 65.45 | 99.44 |
| Ta4 | 93,025 | 92,080 | 91,602 | 91,602 | 18,990,418 | 207 | 98.27 | 94.85 | 65.34 | 98.47 |
| Tc4 | 99,548 | 99,319 | 98,895 | 98,895 | 20,507,558 | 207 | 98.52 | 95.38 | 65.31 | 99.34 |

Ta, tomato seedling stage; Tb, tomato flowering stage; Tc, tomato maturity stage. Ca, cucumber seedling stage; Cb, cucumber flowering stage; Cc, cucumber maturity stage. Sa, soybean seedling stage; Sb, soybean flowering stage; Sc, soybean maturity stage.
