# Supplementary material for: Metagenomic analysis of the dynamical conversion of photosynthetic bacterial communities in different crop fields over different growth periods
Source: PLoS One. 2022 Jul 14;17(7):e0262517. doi: 10.1371/journal.pone.0262517 (PMC9282544; doi:10.1371/journal.pone.0262517)
Supplement: Attachment — Submitted filename: Reviewed by Hafeez.docx [file pone.0262517.s002.docx]

The manuscript by Ju-E et al. provides relevant information on changes in bacterial communities within different crop field caused by the different growth periods. However, substantial revision is needed to improve the clarity of the information provided, particularly regarding to the materials and methods, results, and discussion. Additionally, the manuscript should be proofread to improve the quality of the writing. More specific comments are provided below. Please proofread the manuscript to eliminate typos, misplaced words, missing spaces, sentences that need to be restructured to avoid confusion and improve coherence, and formatting errors.

The title of the manuscript is not very clear, it should be like “Metagenomic analysis of dynamical conversion of photosynthetic bacterial communities in different crop fields over different growth periods”.

Page 2, line 33. Please consider to remove “and” from sentence. different 33 crop species and at different growth stages.

Page 2, line 38. Consider to remove “and” among plant growth and development.

Page 3, line 49. “proteobacteria” Be consistent with the use of bacterial name in italic and with the use of capital letters. Check throughout the manuscript.

Page 4, line 82. Add “help to identify” instead of “help reveal”.

Page 5, line 88 and 89. These two sentences are useless in the start, adjust it in the last before DNA isolation and consider it like “a total of 35 samples were collected from leaves of three crops at different growth stages for subsequent microbe isolation and high throughput sequencing process.

Page 5, line 95. The methodology part is lack of crop plantation points, in which base the block were randomly selected? Fertilization/nutrients rate was same in every block? How many seed were cultivated in every block? Was the seed quantity was varied or same per block? After how much time the samples were collected?

Page 5, line 104. This sentence is unclear, please consider to rewrite it.

Page 8, line 151. Consider to mention the quality filtration information of all the 35 samples as a supplementary table.

Page 11, line 223-226. Fig 3C doesn’t representing the mentioned bacterium except few one.

Page 11, line 227. Explain all the significantly diverse bacterial species among different crop at different growth stages.

Page 15, line 269. Table 5, Consider to mentioned all the a, b, c (P-value) along with the tables. Check with every table showing statistical data.

Page 17, line 294-296. The sentence should be like, the results showed that the photosynthetic bacterial community was common in the phyllosphere and the composition of photosynthetic bacterial community at the phylum and genus levels were similar among the growth stages of the three crops.
