# Supplementary material for: Metagenomic analysis of the dynamical conversion of photosynthetic bacterial communities in different crop fields over different growth periods
Source: PLoS One. 2022 Jul 14;17(7):e0262517. doi: 10.1371/journal.pone.0262517 (PMC9282544; doi:10.1371/journal.pone.0262517)
Supplement: Attachment — Submitted filename: Response to Reviewers.docx [file pone.0262517.s003.docx]

**Manuscript ID:** PONE-D-21-13677

**Title:** Dynamical conversion of photosynthetic bacterial communities in tomato, cucumber, and soybean fields over different growth periods characterized by high-throughput sequencing

**Journal:** PLOS ONE

**Response to Reviewers' comments**

Dear Editor,

We thank you for your careful consideration of our manuscript. We appreciate your response and overall positive initial feedback and made modifications to improve the manuscript. After carefully reviewing the comments made by the Reviewers, we have modified the manuscript to improve the presentation of our results and their discussion, therefore providing a complete context for the research that may be of interest to your readers.

We hope that you will find the revised paper suitable for publication, and we look forward to contributing to your journal. Please do not hesitate to contact us with other questions or concerns regarding the manuscript.

Best regards,

**Journal Requirements**

*1. Please ensure that your manuscript meets PLOS ONE's style requirements, including those for file naming. The PLOS ONE style templates can be found at*

*https://journals.plos.org/plosone/s/file?id=wjVg/PLOSOne_formatting_sample_main_body.pdf and https://journals.plos.org/plosone/s/file?id=ba62/PLOSOne_formatting_sample_title_authors_affiliations.pdf*

**Response:** We thank the Journal. The manuscript was revised accordingly.

*2. We note that the grant information you provided in the ‘Funding Information’ and ‘Financial Disclosure’ sections do not match.*

*When you resubmit, please ensure that you provide the correct grant numbers for the awards you received for your study in the ‘Funding Information’ section.*

**Response:** It was corrected.

*3. We note that you are reporting an analysis of a microarray, next-generation sequencing, or deep sequencing data set. PLOS requires that authors comply with field-specific standards for preparation, recording, and deposition of data in repositories appropriate to their field. Please upload these data to a stable, public repository (such as ArrayExpress, Gene Expression Omnibus (GEO), DNA Data Bank of Japan (DDBJ), NCBI GenBank, NCBI Sequence Read Archive, or EMBL Nucleotide Sequence Database (ENA)). In your revised cover letter, please provide the relevant accession numbers that may be used to access these data. For a full list of recommended repositories, see http://journals.plos.org/plosone/s/data-availability#loc-omics or http://journals.plos.org/plosone/s/data-availability#loc-sequencing*

**Response:** We thank the Journal. In this study, all fastq files are available from the NCBI database (bacterial sequences SRA: SRP193589; BioProject: PRJNA533201; BioSample: SAMN11444424-SAMN11444390; URL: https://www.ncbi.nlm.nih.gov/bioproject/533201).

*4. PLOS requires an ORCID iD for the corresponding author in Editorial Manager on papers submitted after December 6th, 2016. Please ensure that you have an ORCID iD and that it is validated in Editorial Manager. To do this, go to ‘Update my Information’ (in the upper left-hand corner of the main menu), and click on the Fetch/Validate link next to the ORCID field. This will take you to the ORCID site and allow you to create a new iD or authenticate a pre-existing iD in Editorial Manager. Please see the following video for instructions on linking an ORCID iD to your Editorial Manager account:* *https://www.youtube.com/watch?v=_xcclfuvtxQ*

**Response:** We provided the ORCID numbers.

*5. Please review your reference list to ensure that it is complete and correct. If you have cited papers that have been retracted, please include the rationale for doing so in the manuscript text, or remove these references and replace them with relevant current references. Any changes to the reference list should be mentioned in the rebuttal letter that accompanies your revised manuscript. If you need to cite a retracted article, indicate the article’s retracted status in the References list and also include a citation and full reference for the retraction notice.*

**Response:** The reference list was verified.

**Reviewer #1**

*1. Summary: Photosynthetic bacteria are an important component of the phyllosphere, as they play roles in plant health, plant function, and disease suppression. Though there are potential applications for agricultural production, many photosynthetic bacterial communities in the phyllosphere have not been characterized. The authors hypothesize that photosynthetic bacteria are common in the phyllosphere and that the growth period of crops will affect community composition. The results are in support of this hypothesis, as 3,150,689 quality-filtered reads were found in 6725 identified OTUs across 35 pooled samples, alpha diversity changed throughout the growth stages of tomato, cucumber, and soybean, and community dissimilarity was affected by plant growth stage. The reviewer finds the manuscript to be technically sound and that the data supports the authors' conclusions.*

*2. Figures, tables, and captions: The figures are clear and easy to understand, as are the captions.*

**Response:** We thank the Reviewer for taking the time to review our work and for the comments.

*4. There are no issues with the writing that would detract from understanding the work, save for one instance where clarity is needed.*

*Lines 50 - 51: This sentence could be reworded; it reads like it is missing a phrase. As written, this sentence implies that photosynthetic bacteria have only been in the phyllosphere as of recently. Is the sentence meant to convey that the existence of photosynthetic bacteria in the phyllosphere was recently confirmed?*

*Line 54: The phrase "trigger inducible" seems redundant. For example, the literature cited uses the phrase "induces systemic resistance" in the title.*

**Response:** We thank the Reviewer. The entire manuscript was proofread, including the two statements mentioned above.

**Reviewer #2**

*The title of the manuscript is not very clear, it should be like “Metagenomic analysis of dynamical conversion of photosynthetic bacterial communities in different crop fields over different growth periods”.*

**Response**: We thank the Reviewer for the comment. The title was revised as suggested.

*Page 2, line 33. Please consider to remove “and” from sentence. different 33 crop species and at different growth stages.*

**Response**: It was corrected.

*Page 2, line 38. Consider to remove “and” among plant growth and development.*

**Response**: It was corrected.

*Page 3, line 49. “proteobacteria” Be consistent with the use of bacterial name in italic and with the use of capital letters. Check throughout the manuscript.*

**Response**: It was corrected. The names were verified.

*Page 4, line 82. Add “help to identify” instead of “help reveal”.*

**Response**: It was corrected.

*Page 5, line 88 and 89. These two sentences are useless in the start, adjust it in the last before DNA isolation and consider it like “a total of 35 samples were collected from leaves of three crops at different growth stages for subsequent microbe isolation and high throughput sequencing process.*

**Response**: It was corrected as suggested.

*Page 5, line 95. The methodology part is lack of crop plantation points, in which base the block were randomly selected? Fertilization/**nutrients rate was same in every block? How many seed were cultivated in every block? Was the seed quantity was varied or same per block? After how much time the samples were collected?*

**Response**: We thank the Reviewer for the comment. The experiment was conducted from May to August 2018 at the experimental farm of the Hunan Academy of Agricultural Sciences, Changsha, Hunan Province, China (28.22oN, 113.26oE). Tomato cv. Zuan-hong-meili (XinShu Seed Co., Ltd., Beijing, China), cucumber cv. Shuyan 10 (XinShu Seed Co., Ltd., Beijing, China), and soybean cv. Su-qing III (Nanjing Ideal Seedings Co., Ltd., Nanjing, China) were selected for the study. A large block was randomly picked from the experimental farm. Then it was divided into nine small blocks. The size of each block was approximately 40-50 m2, and the nutrients were consistent. Then, 300 plants were planted in each block of cucumber, tomato, and soybean. Each crop was planted in three randomly selected blocks on May 15, 2018 and sampled at the seedling (June 15, 2018), flowering (July 15, 2018), and mature (August 25, 2018) growth stages. This information was added to the Methods.

*Page 5, line 104. This sentence is unclear, please consider to rewrite it.*

**Response**: The sentence was revised.

*Page 8, line 151.* *Consider to mention* *the quality filtration information of all the 35 samples as a s**upplementary table.*

**Response**: We thank the Reviewer. We now present the quality filtration process in Supplementary Table S1.

*Page 11, line 223-226. Fig 3C doesn’t representing the mentioned bacterium except few one.*

**Response**: We thank the Reviewer. We revised the description.

*Page 11, line 227. Explain all the significantly diverse bacterial species among different crop at different growth stages.*

**Response:** We thank the Reviewer. We revised the description.

*Page 15, line 269. Table 5, Consider to mentioned all the a, b, c (P-value) along with the tables. Check with every table showing statistical data.*

**Response:** We thank the Reviewer. We verified all tables. Groups with different superscript letters are significantly different (P<0.05).

*Page 17, line 294-296. The sentence should be like, the results showed that the photosynthetic bacterial community was common in the phyllosphere and the composition of photosynthetic bacterial community at the phylum and genus levels were similar among the growth stages of the three crops.*

**Response**: We thank the Reviewer. The sentence was revised as suggested.
